# Making the most of cataract surgery in patients with diabetes

**Published:** 2019-02-10

**Authors:** Tunde Peto, Frank Sandi, Vineeth Kumar

**Affiliations:** 1 Professor of Clinical Ophthalmology: Queen's University Belfast, Institute of Clinical Sciences Building A, Belfast, Ireland, UK.; 2 Ophthalmologist and Clinical Instructor: The University of Dodoma, College of Health Sciences, Tanzania.; 3 Consultant Ophthalmologist and Vitreoretinal Surgeon: Wirral University Teaching Hospital NHS trust, Wirral, UK.

**Cataract surgery can influence the progression of diabetic eye disease, but may be necessary for treatment and to help the person function effectively. Here is how to make the most of it.**

**Figure:**
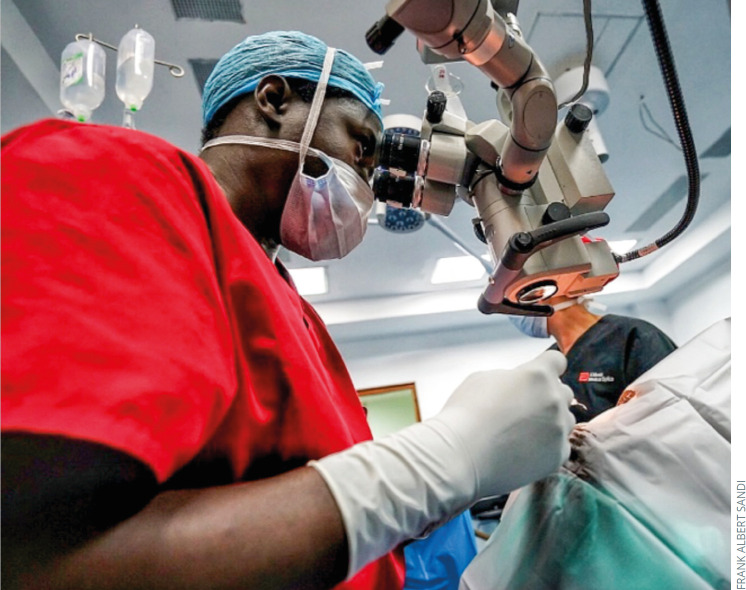
Operating on a cataract patient in Dodoma. **TANZANIA**

Diabetes mellitus is a disease of elevated blood sugar due to insufficient *production* (type 1) or *action* (type 2) of insulin. Type 1 diabetes requires insulin injections, whereas type 2 is managed with diet, exercise, tablets or insulin injections, if needed.

The impact of cataract is twofold:

1. Poor view of the retina means that laser and intravitreal injections to treat the visual complications of diabetes, such as diabetic retinopathy or maculopathy, might become impossible. Without treatment, these can progress to the point where potential vision is so poor that cataract removal will not lead to visual improvement.
2. Failing sight, due to cataract, cause practical difficulties with the timely administration of medication to control blood glucose (blood sugar). This is especially true for those who have to inject insulin.

Unfortunately, cataract tends to have an early onset in those with diabetes and progresses rapidly if blood sugar control is suboptimal. This is most likely due to the osmotic changes taking place within the lens, usually resulting in cortical or posterior subcapsular lens opacities.

## Identifying patients with diabetes

In low- and middle-income countries, the first time many people are recognised as having diabetes is when they come to an eye clinic for cataract surgery. It is therefore vital to take a careful history, conduct a thorough eye examination and measure blood glucose before surgery to identify those who may have diabetes.

People who are young and have both diabetes and cataract may have neglected their blood sugar control over the years or may not have had access to facilities to enable them to achieve good control. If blood sugar is not controlled, it will be very difficult to prevent vision loss to diabetic retinopathy.

## Referral

Cataract patients with diabetes must be referred to an endocrinologist or suitably qualified physician so their diabetes can be managed.

Ensure that the endocrinologist or physician knows where to refer people with diabetes for regular retinal examinations, during which an eye care professional will check for signs of diabetic eye disease (diabetic retinopathy and diabetic maculopathy).

## The risks of cataract surgery

There is a potential risk that diabetic eye disease may progress as a direct result of cataract surgery. This depends on:

- The level of retinopathy and maculopathy at the time of surgery
- Whether surgery was complicated or uncomplicated
- Blood sugar control.

All these factors have an impact on the progression of retinopathy. Squirrell et al^1^ noted that much of the progression of retinopathy in their cohort was due to natural history and that uncomplicated cataract surgery using phacoemulsification is not an independent factor for accelerated progression of retinopathy after surgery. **Editor's note:** the study did not compare phacoemulsification with small-incision cataract surgery.

Zaczek et al^2^ and the UK EMR observational study^3^ found that eyes with pre-existing macular oedema had the worst prognosis for visual rehabilitation. If the fundus is visible prior to surgery and there is diabetic retinopathy and/or maculopathy, it is worth following the treatment guidelines in Table 1 prior to cataract surgery in order to prevent worsening of the diabetic retinopathy.

**Table 1:** Treatment of cataract surgery patients who have diabetes.

Diagnosis	Management
No diabetic retinopathy (DR)	Discharge to annual screening
Mild, non-proliferative DR (NPDR)	Discharge to annual screening or observe in clinic
Severe NPDR	Pan-retinal photocoagulation laser or anti-VEGF injections (when patients have to travel a long way), otherwise observe in clinic and perform fundus fluorescein angiography
Proliferative DR	Pan-retinal photocoagulation laser or anti-VEGF
Diabetic macular oedema (DMO) and NPDR	Anti-VEGF with or without pan-retinal photocoagulation laser
DMO + PDR	Anti-VEGF and pan-retinal photocoagulation laser
Diabetic maculopathy	NSAIDS or pan-retinal photocoagulation laser or anti-VEGF or steroids

## Communicating with patients

It is important to manage the expectations of cataract patients who also have diabetes, as potential visual loss due to diabetic eye disease may affect the amount of visual improvement possible after surgery.

Talking with patients about all the risks and benefits of treatment is an important part of the process, both preoperatively and postoperatively, as it helps both parties to clearly understand the potential visual outcomes of the operation. Discuss the potential need for postoperative interventions, such as laser treatment and/or anti-VEGF injections, before taking consent. It is important to ensure that the patient is willing, and able, to come back to the clinic for these treatments.

In patients with more advanced disease, ask what treatment they have undergone; e.g., laser treatment, anti-VEGF injections, or intravitreal steroids; this can also affect outcomes.

From a medico-legal perspective, it is important to document all of the above information carefully.

You can improve patients' experience by giving clear instructions about how to care for their eye after surgery and when to come back. Put in place specific, identifiable personnel as a point of contact for patients. This will minimise the loss of patients at postoperative and subsequent follow-up visits.

## Blood sugar control

There have always been questions about optimum blood sugar control for patients with diabetes prior to cataract surgery. However, there is no published evidence to suggest that elevated blood glucose level at the time of surgery has a negative effect on outcome,^4^ and the risk of cancelling a patient (who may not return) may outweigh the benefits – if any exist – of deferring surgery until they control their blood sugar better.

It is not unreasonable however, to take steps to reduce blood sugar (e.g. giving an additional insulin injection if they have Type 1 diabetes) on the day of surgery if patients present with very high levels.^5^

## Intraoperative considerations

Cataract surgery in patients with diabetes can be complicated as a direct result of their diabetes. There may be poor pupil dilation due to lack of tone in the iris muscles, secondary to autonomic neuropathy; this is often seen in patients with chronic diabetes. If there is poor dilation, or in patients with intraoperative miosis, it is necessary to manually dilate the pupil, whether by using pupil hooks/dilators or by performing an iridectomy (see pp. 84–85).

In younger people with diabetes and cataract, extra care must be taken as their capsule tends to be elastic, leading to complications such as capsular tears. Use trypan blue ophthalmic solution to stain the anterior lens capsule.

Postoperative inflammation is expected to be worse in patients with diabetes. This can lead to contraction of the anterior capsule and phimosis, which in turn limits the view of the peripheral retina after surgery.

Capsulorrhexis should therefore be large, which means the optic should be at least 6 mm in size.

## Postoperative care

Ideally, patients with diabetes who have recently undergone cataract surgery should undergo a careful eye examination, including a detailed fundus examination, to check for excessive inflammation and signs of diabetic eye disease. Offer treatment as shown in Table 1.

## Discussion

Cataract surgery in patients with diabetes is a complex subject. The level of retinopathy at the time of surgery, complicated or uncomplicated surgery and diabetic control all have a potential impact on the progression of diabetic eye disease.

Despite these risks, cataract surgery may be necessary to allow the person to function effectively and improve their adherence to their prescribed diabetic medication regimen. Surgery may also be necessary in order to make it possible to treat diabetic eye disease using pan-retinal photocoagulation laser or other treatment options.
